# Analyses of MiRNA Functions in Maize Using a Newly Developed ZMBJ-CMV-2b_N81_-STTM Vector

**DOI:** 10.3389/fpls.2019.01277

**Published:** 2019-10-17

**Authors:** Xuedong Liu, Sijia Liu, Rong Wang, Xi Chen, Zaifeng Fan, Boming Wu, Tao Zhou

**Affiliations:** ^1^State Key Laboratory for Agro-Biotechnology and Key Laboratory for Pest Monitoring and Green Management-Ministry of Agriculture and Rural Affairs, Department of Plant Pathology, China Agricultural University, Beijing, China; ^2^Institute of Medicinal Plant Development, Chinese Academy of Medical Sciences and Peking Union Medical College, Beijing, China

**Keywords:** plant endogenous microRNAs, virus-based miRNA suppression, cucumber mosaic virus, high-throughput, miRNA function

## Abstract

Endogenous microRNAs (miRNAs) play pivotal roles in plant development and responses to various biotic or abiotic stresses. Up to now, more than 500 maize miRNAs have been identified. However, functions of these identified miRNAs remained largely unknown due mainly to the lack of rapid and reliable tools. We previously reported a cucumber mosaic virus strain ZMBJ (ZMBJ-CMV)-based gene silencing vector for rapid and efficient gene function studies in maize lines with agronomical importance. Because ZMBJ-CMV induces very mild disease symptoms but strong gene silencing in maize, we decided to further modify this vector to suppress miRNA expressions in maize. The newly developed ZMBJ-CMV-2b_N81_-STTM vector expresses a short tandem target mimic (STTM) containing two target-mimic sequences separated by a short spacer sequence. Our results showed that ZMBJ-CMV-2b_N81_-STTM can be used to investigate miRNA function in *Nicotiana benthamiana* and maize seedlings. The ZMBJ-CMV-2b_N81_-STTM-based downregulation of Nbe-miR165/166 or Nbe-miR159 induced specific and strong miRNA-sequestering phenotypes, and increased the expressions of their predicted target genes. For maize, the ZMBJ-CMV-2b_N81_-STTM based downregulation of zma-miR167 or zma-miR482 caused a decrease of lateral roots growth and a plant stunting phenotypes, respectively. In both cases, the target genes of zma-miR167- or zma-miR482 were increased significantly. Thus, we consider ZMBJ-CMV based VbMS system as a useful tool for high-throughput investigations of miRNA functions in maize.

## Introduction

Maize (*Zea mays*) is one of the most important cereal crops worldwide. It can be used for human consumption, animal feed and biofuel production (Schnable et al., 2009). To improve maize growth and production, substantial efforts have been made to identify the functions of maize proteins, genes and microRNAs (Lagrimini, 2018). microRNAs (miRNAs) are a class of endogenous 20–24 nucleotide (nt) small noncoding RNAs that can regulate gene expressions through directing the RNA-induced silencing complex to cleave mRNA or through translational repression or, likely, DNA methylation (Song et al., 2019). miRNAs are known to play important roles in regulation of plant development and growth, and responses to abiotic and/or biotic stresses (Sunkar et al., 2012). To date, approximately 502 unique maize miRNAs have been identified and deposited at miRBase (http://www.mirbase.org/release 22.1, October 2018). However, only four identified maize miRNAs, including miR166, miR156, miR172 and miR528 have been functionally characterized (Chuck et al., 2007a; Chuck et al., 2007b; Nogueira et al., 2007; Sun et al., 2018).

Functional studies of miRNAs depends largely on screenings of mutant plants with specific mutagenized miRNAs, through knocking down miRNAs via expressions of target mimics (TM) or short tandem target mimics (STTM), through stable transformation, or through using virus-based miRNA silencing (VbMS) vector (Reyes and Chua, 2007; Yan et al., 2012; Zhang et al., 2017; Peng et al., 2018; Zhang et al., 2018; Wang et al., 2019; Yang et al., 2019). For instance, maize *Corngrass1* (*Cg1*) mutant was found to overexpress two tandem miR156 in meristem tissues and in lateral organs (Chuck et al., 2007a). Maize *tasselseed4* (*ts4*) mutant was determined to express mir172 that can target *APETALA2*, a floral homeotic transcription factor. Maize mutant carrying a *ts4* mutation was shown to promote carpel development on tassels and more meristem branching (Chuck et al., 2007b). Analysis of a collection of *Arabidopsis thaliana* target mimics, Todesco and colleagues reported that the MIM172 plants show late flowering and narrow leaf phenotypes (Todesco et al., 2010). A more recent report indicated that knockdown of rice microRNA166 through STTM results in morphological changes and drought resistance in rice (Zhang et al., 2018).

Earlier studies on miRNA functions were done through expressing target mimics (TM) using an *IPS1* (*INDUCED BY PHOSPHATE STARVATION1*)-derived structure containing a three-nucleotide interruption linker in the miRNA cleavage site. This structure could be used as a target mimic to sequester miRNAs of interest (Franco-Zorrilla et al., 2007). Unfortunately, TM constructs could not be used to induce efficient silencing of some highly abundant miRNAs and produce clear phenotypes (Todesco et al., 2010). Recently, a short tandem target mimic (STTM) consisting of two TMs separated by a 48–88 nt stem-loop like linker sequence was developed (Yan et al., 2012). With better and more specific binding between miRNAs and the mimics, suppressions of miRNA activities through STTM were much more efficient than that induced by regular TMs (Todesco et al., 2010; Yan et al., 2012).

More recently, several virus-based miRNA suppression (VbMS) systems have been developed to express STTM in plant. These VbMS systems were shown to have efficient miRNA suppression abilities and are simple to use. For example, four plant viruses: tobacco rattle virus (TRV), cotton leaf crumple virus (CLCrV), cucumber mosaic virus LS strain (LS-CMV) and potato virus X (PVX) had been modified to express STTMs to silence miRNAs. These systems were shown to be effective in Arabidopsis, cotton, *Nicotiana benthamiana* and/or tomato, respectively (Gu et al., 2014; Sha et al., 2014; Liao et al., 2015; Zhao et al., 2016). For monocotyledonous plants, only barley stripe mosaic virus (BSMV)-based VbMS system had been reported for wheat (Jiao et al., 2015; Jian et al., 2017). We speculate that to conduct investigations on miRNA functions in more cereal crops, including maize, more efficient and reliable VbMS systems are needed.

We previously reported ZMBJ-CMV as a maize-infecting strain of CMV (Wang et al., 2013). We then reported that ZMBJ-CMV had been modified to serve as an efficient virus-induced gene silencing (VIGS) vector. Using this VIGS vector, strong gene silencing phenotypes were observed in 18 agronomically important maize inbred lines, including the model line B73 (Wang et al., 2016). Because the ZMBJ-CMV-based vector causes mild infection symptoms in maize and can induce efficient gene silencing in maize (Wang et al., 2016), we decided to further modify the RNA2 cDNA plasmid of ZMBJ-CMV to express STTM in plant. Using this newly developed ZMBJ-CMV VbMS system, we successfully downregulated two endogenous miRNAs in *N. benthamiana* and two endogenous miRNAs in B73 maize plants. We consider that this VbMS system is useful for routine and fast analyses of miRNA functions in maize.

## Materials and Methods

### Plasmid Construction

ZMBJ-CMV plasmid pCMV101, pCMV301 and pCMV201-2b_N81 _were described previously (Wang et al., 2016). A 48 nt oligonucleotide was synthesized and used as a template for further STTM PCR amplifications. A three nucleotide (nt) sequence (CTA) was inserted into various STTM molecules at the site corresponding to the nucleotide position 10–11 of the targeted miRNA sequences as reported (Yan et al., 2012). All the STTMs were then amplified using STTM specific primers plus 15 additional nts homologue to the vector sequences (**Supplementary Table 1**) using the Fast*Pfu* polymerase (TransGen Biotech). The resulting STTM fragments were digested with restriction enzyme *Kpn*I and *Xba*I, and cloned individually into the *Kpn*I/*Xba*I site in pCMV201-2b_N81 _using the In-Fusion HD Cloning kit as instructed (Takara Bio).

### Plant Culture and Growth Condition

*N. benthamiana* plants were grown in pots inside growth chambers set at 24/22°C (day/night), 16/8 h (light/dark) and 60% relative humidity.

Maize seeds (inbred line B73) were obtained from the National Maize Improvement Center, China Agricultural University, Beijing, China. All seeds were germinated on wet blotting papers inside petri dishes at 25°C and in the dark. The germinated seeds were transplanted into pots and then grown inside growth chambers set at 20/18°C (day/night) and 16/8 h (light/dark).

In other experiments, the germinated seeds were grown in hydroponic culture media with continuous aeration as described (Yu et al., 2014; Pan et al., 2016). The nutrient solution consisted 0.65 mM MgSO_4_, 2 mM NH_4_NO_3_, 2 mM CaCl_2_, 0.75 mM K_2_SO_4_, 0.1 mM KCl, 0.25 mM KH_2_PO_4_, 0.2 mM Fe-EDTA, 1 × 10^−3^ mM MnSO_4_, 1 × 10^−3^ mM ZnSO_4_, 1 × 10^−4^ mM CuSO_4_, 5 × 10^−6^ mM (NH_4_)_6_Mo_7_O_24_, and 1 × 10^−3^ mM H_3_BO_3_, pH 5.8–6.0.

### Plant Inoculation and VbMS Assay

For ZMBJ-CMV-based VbMS, plasmid pCMV101, pCMV301, pCMV201-2b_N81_-STTM and its derivatives, were introduced individually into *Agrobacterium tumefaciens* strain C58C1. Preparations of *A. tumefaciens* cultures and infiltration of *N. benthamiana *leaves were same as described by Wang et al. (2016). At least six plants were used each for VbMS construct and each treatment was repeated three times. The infiltrated plants were monitored for symptom development starting from 7 days post infiltration (dpi).

For maize inoculation, the agro-infiltrated *N. benthamiana* leaves were harvested at 3 dpi and then ground in 0.1 M phosphate buffer, pH 7.0. After 3 min centrifugation at 4,000 rpm and at 4 °C, supernatant of each sample was used to inoculate maize seeds using the vascular puncture method (Louie, 1995; Redinbaugh et al., 2001). The inoculated seeds were germinated at 25 °C and in the dark for 2 days. The germinated seeds were transplanted into soil in pots and then grown inside the growth chambers as described above. For each VbMS treatment, 150 to 200 seeds were inoculated and the experiment was repeated three times. Virus infection in each plant was confirmed via symptom observation and RT-PCR detection.

### RNA Isolation and RT-PCR Analysis

The 2^nd^ upmost young leaves of *N. benthamiana* plants agro-inoculated with the ZMBJ-CMV-STTM159 or ZMBJ-CMV-STTM165/166 or the first true leaves of maize plants VPI-inoculated with the ZMBJ-CMV-STTM167 or ZMBJ-CMV-STTM482 were harvested at 14 dpi. Total RNA was isolated from individual harvested leaf samples using TRIzol reagent as instructed (TIANGEN). After RNase-free DNase I (Takara Bio) treatment, 2 μg total RNA was used in each first-strand complementary DNA synthesis together an oligo(dT) primer or CMV-specific primers, and the Moloney murine leukemia virus reverse transcriptase (Promega Corporation).

### Quantitative Reverse Transcription Polymerase Chain Reaction (RT-qPCR)

RT-qPCR was performed using a FastSYBR mixture (Beijing ComWin Biotech). Expressions of *EIF4a* and *ZmUbi* were used as internal controls for *N. benthamiana* and maize, respectively. Stem-loop RT-qPCR was performed as described (Varkonyi-Gasic, 2017). The miRNA complementary regions in various reverse transcription primers were extended to contain the cleavage site to avoid STTM contamination. Statistical differences between the means of treatments were determined using the Student’s *t* test. Each experiment was replicated at least three times.

Sequences of miRNAs described in this article were retrieved from the GenBank (https://www.ncbi.nlm.nih.gov/) under the following accession numbers: *ZmARF3* (GRMZM2G078274); *ZmARF9* (GRMZM2G073750); *ZmARF16* (GRMZM2G028980); *ZmARF18* (GRMZM2G035405); *ZmARF22* (GRMZM2G089640); *ZmARF30* (GRMZM2G475882); *ZmARF34* (GRMZM2G081158); *ZmIAR3* (GRMZM2G090779); *Z. mays polyubiquitin* (XM_008647047.3); *ZmTPS2* (GRMZM2G079928).

## Results

### Analysis of MiRNA Function Using a ZMBJ-CMV-Based STTM Expression Vector

**Figure 1:**
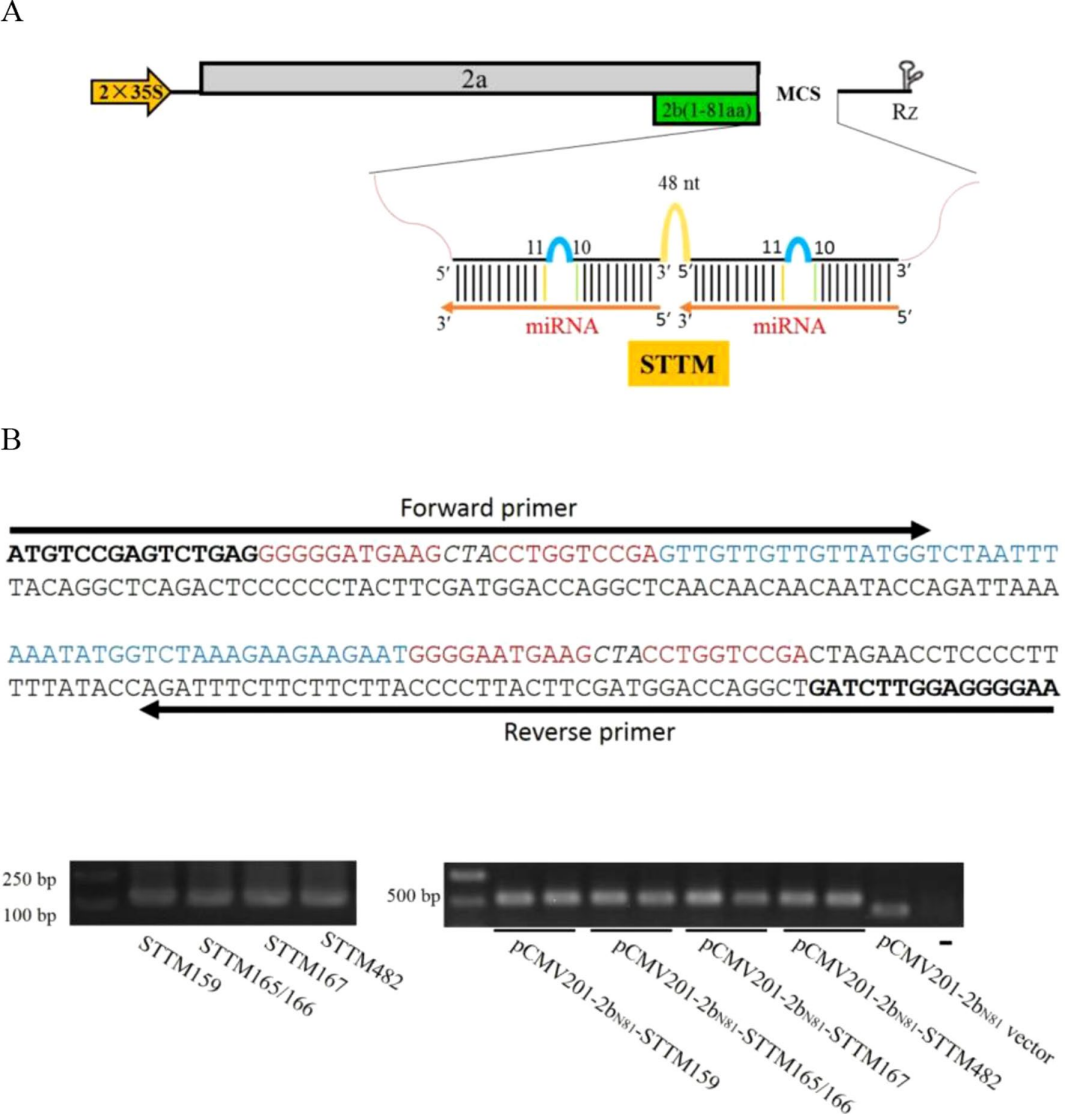
Construction of ZMBJ-CMV201-2b_N81_-STTM. **(A)** Schematic ZMBJ-CMV201-2b_N81_ vector and insertion of STTM. STTM fragments are inserted at the multiple cloning sites (MCS). **(B)** One-step method used to construct a STTM sequence and the recombination-based ligation method. The STTM fragments were PCR amplified with specific forward and reverse primers (**Supplementary Table S1**). Bold, the two adapter sequences; Red, complementary strand of miR165/166; Italicized, a three nucleotide interruption linker sequence; Blue, 48 nt template; Arrows indicate the locations of the forward and reverse primers.

Our previous report showed that ZMBJ-CMV induces very mild symptoms in maize and can cause efficient gene silencing in this host (Wang et al., 2016). The ZMBJ-CMV-based VIGS vector consists of three plasmids: pCMV101, pCMV201-2b_N81_ and pCMV301, expressing ZMBJ-CMV RNA1, RNA2, and RNA3, respectively (Wang et al., 2016). Plasmid pCMV201-2b_N81_ contains a truncated 2b gene encoding the N-terminal 81 amino acids of the 2b protein and can be used to induce efficient gene silencing in maize after being inserted with a 150–300 nt fragment, representing a partial sequence of a target gene. Considering the expression strategy of STTM and the capacity of plasmid pCMV201-2b_N81 _for a foreign inserts, we hypothesized that pCMV201-2b_N81 _could be used to investigate the functions of miRNAs through expressing short tandem target mimic (STTM). In this study, we cloned various STTMs at the multiple cloning sites (MCS) in pCMV201-2b_N81_ (**Figure 1**). A spacer (48 nt) and two target mimics corresponding to a specific miRNA were used to PCR to produce a STTM molecule as described before (Sha et al., 2014). The resulting STTM molecules were individually inserted behind the truncated 2b coding sequence via an In-Fusion clone method (Takara Bio) to produce pCMV201-2b_N81_-STTM159, pCMV201-2b_N81_-STTM165/166, pCMV201-2b_N81_-STTM167, and pCMV201-2b_N81_-STTM482, respectively. These plasmids were then used to express nbe-miR159 STTM or nbe-miR165/166 STTM in *N. benthamiana* or zma-miR167 STTM or zma-miR482 STTM in maize (**Figure 1B**).

### Effective Suppression of Endogenous MiRNA in *N. benthamiana* Using a ZMBJ-CMV-Based VbMs System

**Figure 2:**
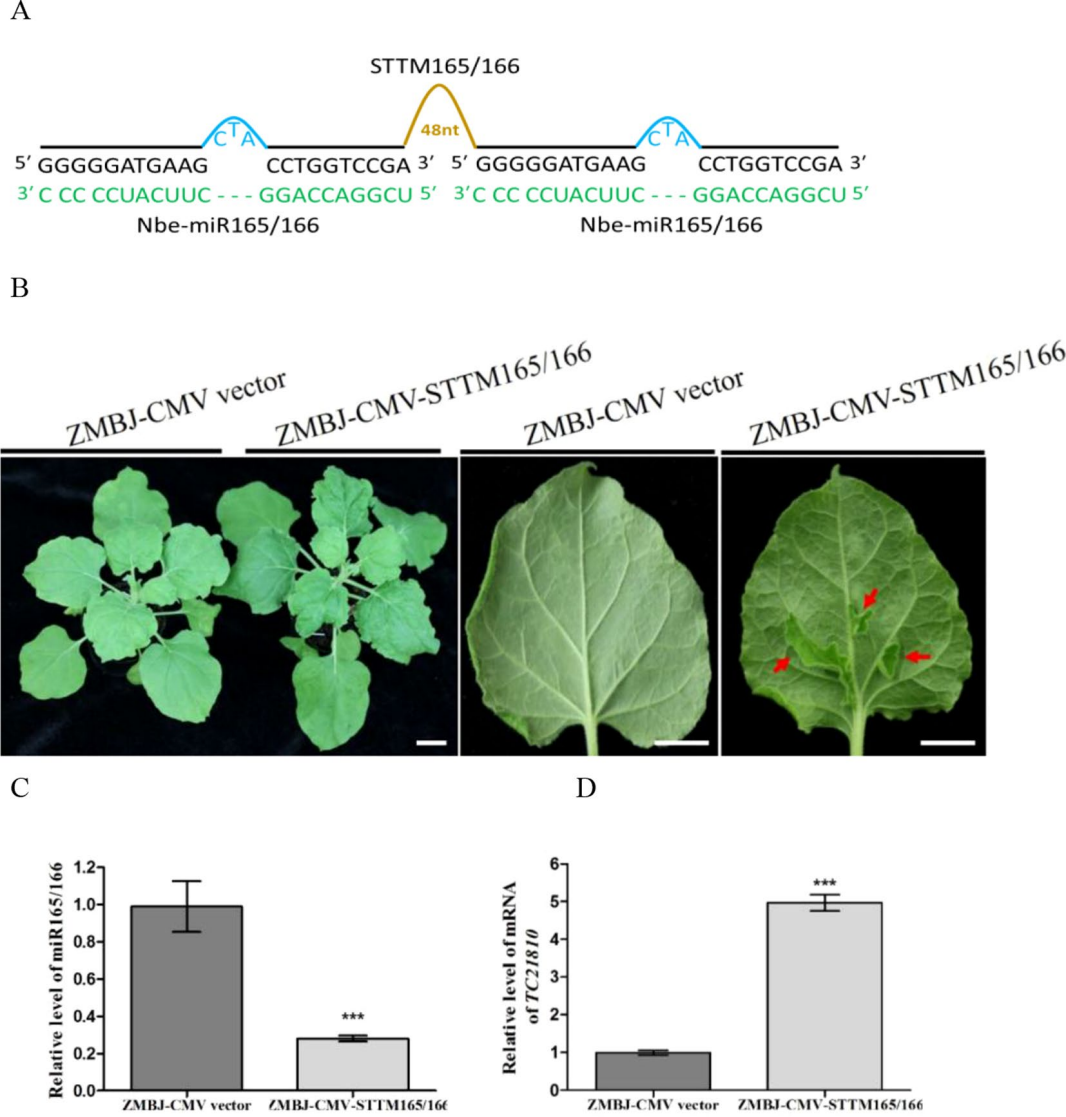
Silencing miR165/166 expression in *Nicotiana benthamiana* using ZMBJ-CMV-STTM165/166. **(A)** A diagram of STTM165/166 insert in the ZMBJ-CMV-based VbMS vector. 48 nt, a 48 nt stem-loop linker; —, no nucleotide at this position. **(B)** Phenotypes of plants infected with ZMBJ-CMV (control) or ZMBJ-CMV-STTM165/166. Photographs were taken at 28 dpi. Images of two detached leaves (abaxial side) are shown on the right side. Red arrows indicate enations on the abaxial leaf side. Bars = 2 cm. **(C)** Relative expression of miR165/166 determined by stem-loop RT-qPCR. **(D)** Relative expression of miR165/166 target gene *TC21810* in the ZMBJ-CMV (control) or ZMBJ-CMV-STTM165/166 infected maize plants. The 2^nd^ upmost young leaves were harvested from the assayed plants at 14 dpi and used for this study. The results were analyzed using the GraphPad Prism 5 software (GraphPad Software, Inc., La Jolla, CA, USA). Each bar represents the mean ± SEM from three independent experiments. Statistical significance between the two treatments was determined by the Student’s *t*-test. ***, p < 0.001.

**Figure 3:**
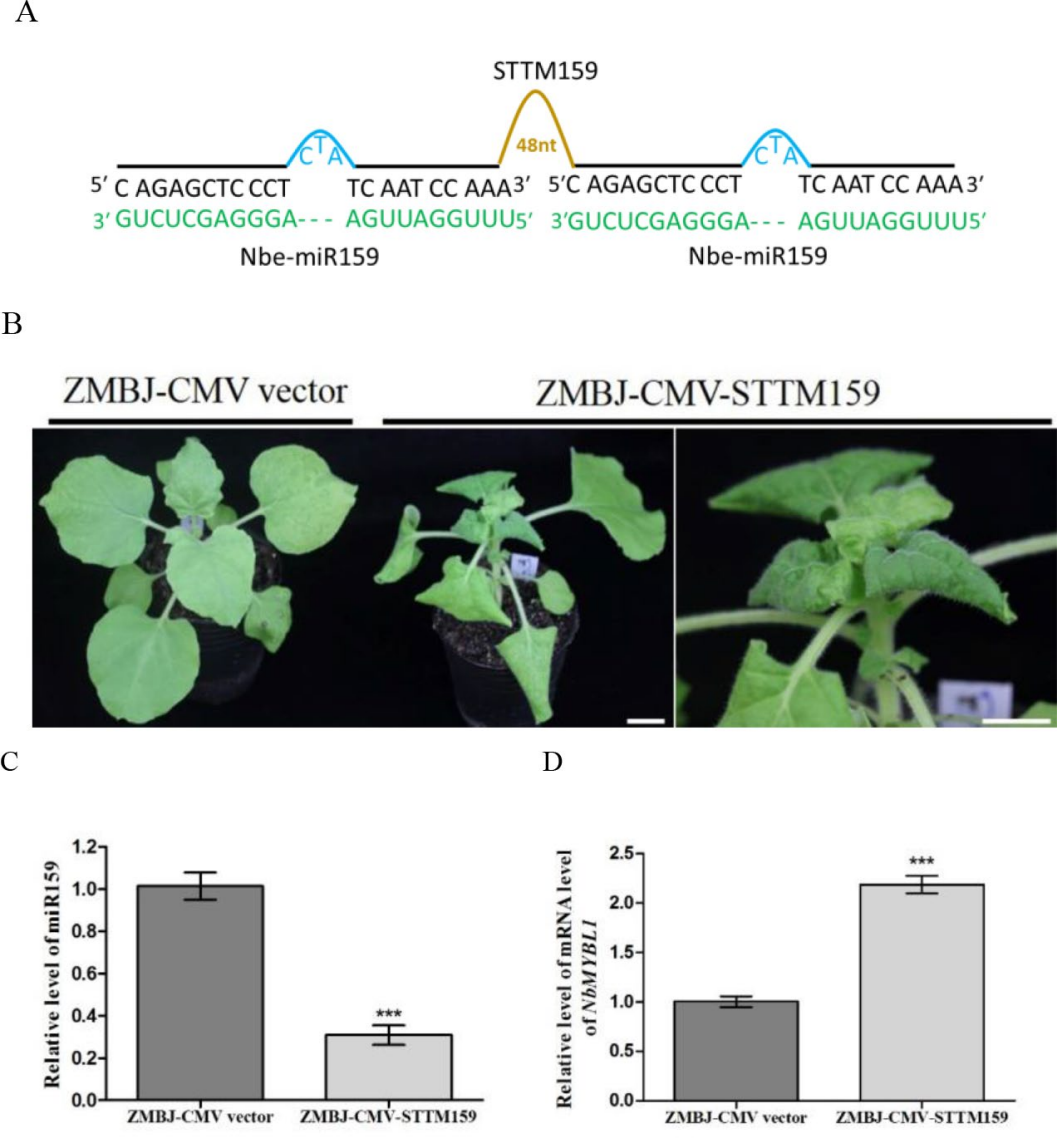
Silencing miR159 expression in *N. benthamiana* using ZMBJ-CMV-STTM159. **(A)** A diagram of the STTM159 insert in the ZMBJ-CMV-based VbMS vector. 48 nt, a 48-nt long stem-loop like linker; —, no nt at this position. **(B)** Phenotypes of *N. benthamiana* plants infected with ZMBJ-CMV (control) or ZMBJ-CMV-STTM159. Photographs were taken at 14 dpi. An enlarged image of the ZMBJ-CMV-STTM159 infected plant is shown at the right side. Bars = 2 cm. **(C)** Relative expression of miR159 in *N. benthamiana* determined by the stem-loop RT-qPCR. **(D)** Relative expression of miR159 target *NbMYBL1* in the ZMBJ-CMV (control) or ZMBJ-CMV-STTM159-infected plants. The 2^nd^ upmost young leaves were harvested from the assayed plants at 14 dpi and used for this study. The results were analyzed and presented as described in **Figure 2**. ***, p < 0.001.

To determine whether ZMBJ-CMV-based VbMS vectors could silence miRNAs in plants, we agro-infiltrated pCMV201-2b_N81_-STTM159 or pCMV201-2b_N81_-STTM165/166 vector into leaves of *N. benthamiana* plants separately. By 14 dpi, we found that expression of miR165/166 and miR159 in agro-infiltrated plants were strongly suppressed (**Figures 2**, **3**).

MiR165/166 was predicted to cleavage class III homeodomain-leucine zipper (HD-ZIP III) transcription factors important for plant shoot apical dominance and leaf polarity (Li et al., 2005; Liu et al., 2009; Merelo et al., 2016). Downregulation of miR165/166 expression using TRV-, PVX- or CMV-LS-based VbMS vector was shown to cause reduction of apical dominance, generations of ectopic tissues from midribs, and pleiotropic developmental defects (Sha et al., 2014; Liao et al., 2015; Zhao et al., 2016). In this study, expression of pCMV201-2b_N81_-STTM165/166 vector in *N. benthamiana* plants also caused strong enation along the veins of leaves and leaf malformation (**Figure 2**). No plant stunting was observed for plants agro-infiltrated with ZMBJ-CMV-STTM165/166 at 28 dpi. In addition, all the infiltrated plants displayed clear leaf developmental defects, demonstrating ZMBJ-CMV-based vector as a high efficient vector of VbMS. RT-qPCR analyses confirmed that the level of nbe-miR165/166 in the ZMBJ-CMV-STTM165/166-infiltrated plants was knocked down about 70% compared with the control plants. Also, the level of *TC21810* mRNA, a HD-Zip gene regulated by miR165/166, was elevated by about five fold (**Figures 2C, D**).

MiR159 was predicted to cleave several *MYB-like transcription factor* genes in *N. benthamiana* (Allen et al., 2007; Palatnik et al., 2007). After nbe-miR159 in *N. benthamiana* plants was downregulated using a PVX-based STTM vector, the plants showed strong stunting and leaf distortion, including smaller and downward-curled leaves, darker green mosaic, and shorter petioles compared with that shown by the PVX infected plants (Zhao et al., 2016). Liao and others reported that miR159 played important roles in CMV-induced symptom development and suppression of miR159 expression using a LS-CMV-based vector caused severe plant stunting and malformation similar to the symptoms induced by the severe Fny-CMV strain (Liao et al., 2015). In our study, infection of ZMBJ-CMV-STTM159 in *N. benthamiana* also induced severe plant stunting and leaf curling by 7 dpi (**Figure 3**). Symptoms caused by ZMBJ-CMV-STTM159 infection in *N. benthamiana* plants were similar to that reported for the PVX-STTM159-infected *N. benthamiana* plants (Zhao et al., 2016). Result of RT-qPCR showed that level of miR159 in the ZMBJ-CMV-STTM159-infected plants was reduced by about 65% while the level of *MYB-like transcription factor* (*NbMYBL1*), a predicted target of miR159, was increased by approximately 2.4 folds compared with the ZMBJ-CMV-infected plants (**Figures 3C, D**).

### Silencing MiR167 Using ZMBJ-CMV-STTM167 Reduced Maize Lateral Root Number and Length

Arabidopsis and soybean miR167 was reported to play critical roles in lateral root development and architecture (Gifford et al., 2008; Todesco et al., 2010; Wang et al., 2015). In maize, miR167 is predicted to cleave several *auxin responsive factors* (*ARF*s) genes (http://wmd3.weigelworld.org/cgi-bin/webapp.cgi). The function of miR167 in maize root development remains unknown.

**Figure 4:**
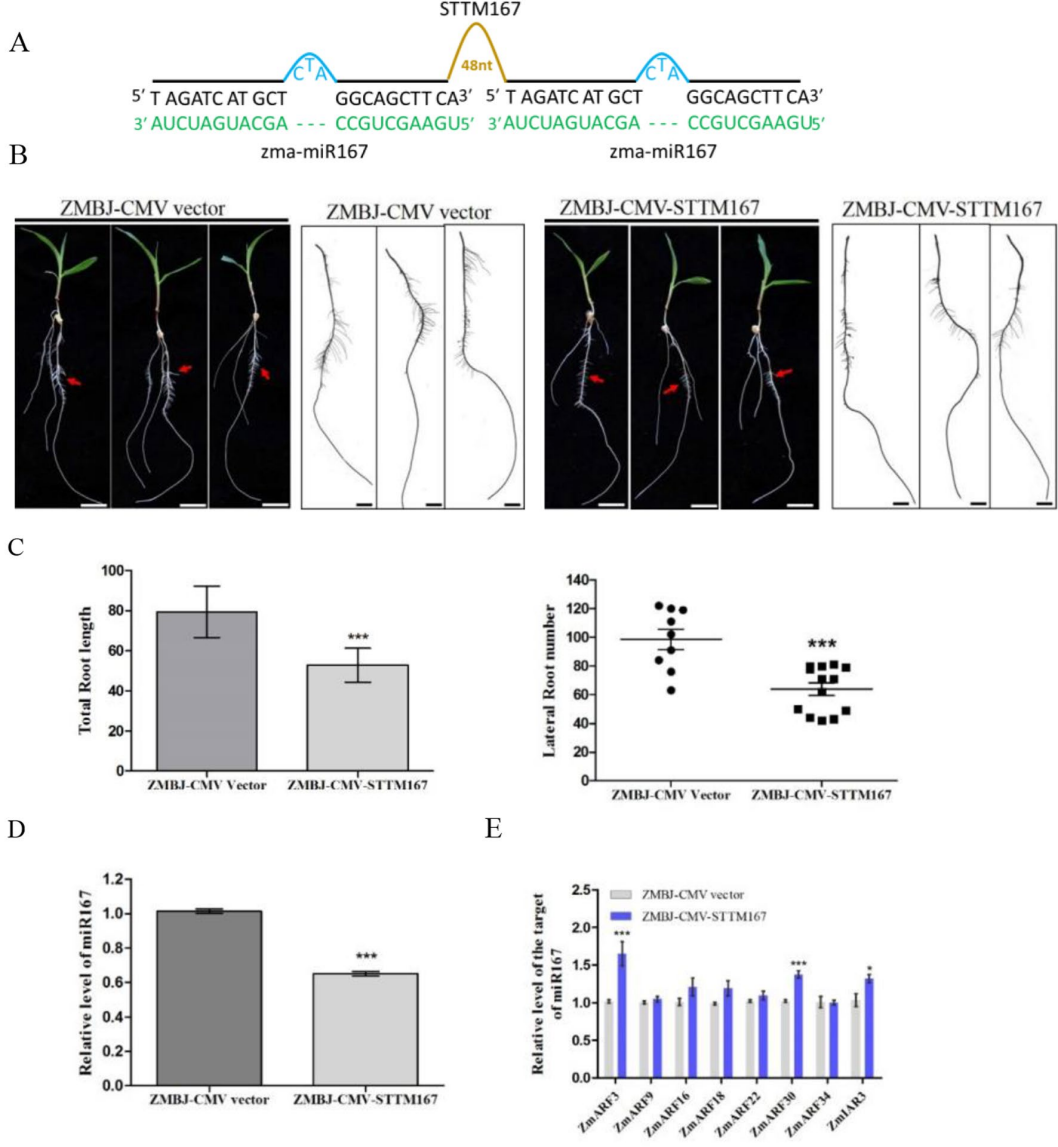
Silencing miR167 expression in maize using ZMBJ-CMV-STTM167. **(A)** A diagram of the STTM167 insert in the ZMBJ-CMV-based VbMS vector. 48 nt, a 48-nt long stem-loop like linker; —, no nt at this position. **(B)** Roots of maize seedlings infected with ZMBJ-CMV (control) or ZMBJ-CMV-STTM167 were photographed at 14 dpi. Bars = 2 cm. **(C)** Measurement of total root length of the ZMBJ-CMV or ZMBJ-CMV-STTM167 infected maize seedlings at 14 dpi. ***, p < 0.001. **(D)** Relative expression of miR167 in the ZMBJ-CMV or ZMBJ-CMV-STTM167 infected maize plants determined by stem-loop RT-qPCR. **(E)** Relative expression of miR167 target genes *ZmARF3*, *ZmARF9*, *ZmARF16*, *ZmARF18*, *ZmARF22*, *ZmARF30*, *ZmARF34* and *ZmIAR3* in the ZMBJ-CMV or ZMBJ-CMV-STTM167 infected maize seedlings. The first true leaves of the assayed plants were harvested at 14 dpi and used for this study. The results were analyzed and presented as described in **Figure 2**. *, p < 0.05; ***, p < 0.001.

In this study, we downregulated Zma-miR167 in maize using ZMBJ-CMV-STTM167 (**Figure 4A**). The assayed plants were grown hydroponically so that root development of these plants could be readily investigated. Observation of symptom and RT-PCR detection confirmed the infection by ZMBJ-CMV vector or ZMBJ-CMV-STTM167 on maize seedlings. Approximately 50–70% of the ZMBJ-CMV-STTM167-infected plants showed significant reduction of lateral root number and length compared with that shown by the ZMBJ-CMV-infected plants by 14 dpi (**Figure 4**). With the tissues showing reduction of lateral root number and length, results of RT-qPCR assays showed that the level of miR167 was decreased significantly in the ZMBJ-CMV-STTM167-infected plants while the levels of miR167 target genes: *auxin response factors* (*ZmARF3* and *ZmARF30*) and *INDOLE-3-ACETIC ACIDALANINE RESISTANT3* (*ZmIAR3*), were up-regulated significantly in the ZMBJ-CMV-STTM167-infected maize plants compared with the ZMBJ-CMV-infected control plants (**Figures 4D, E**).

### Silencing MiR482 in Maize Using ZMBJ-CMV-STTM482 Inhibited Maize Seedling Growth

**Figure 5:**
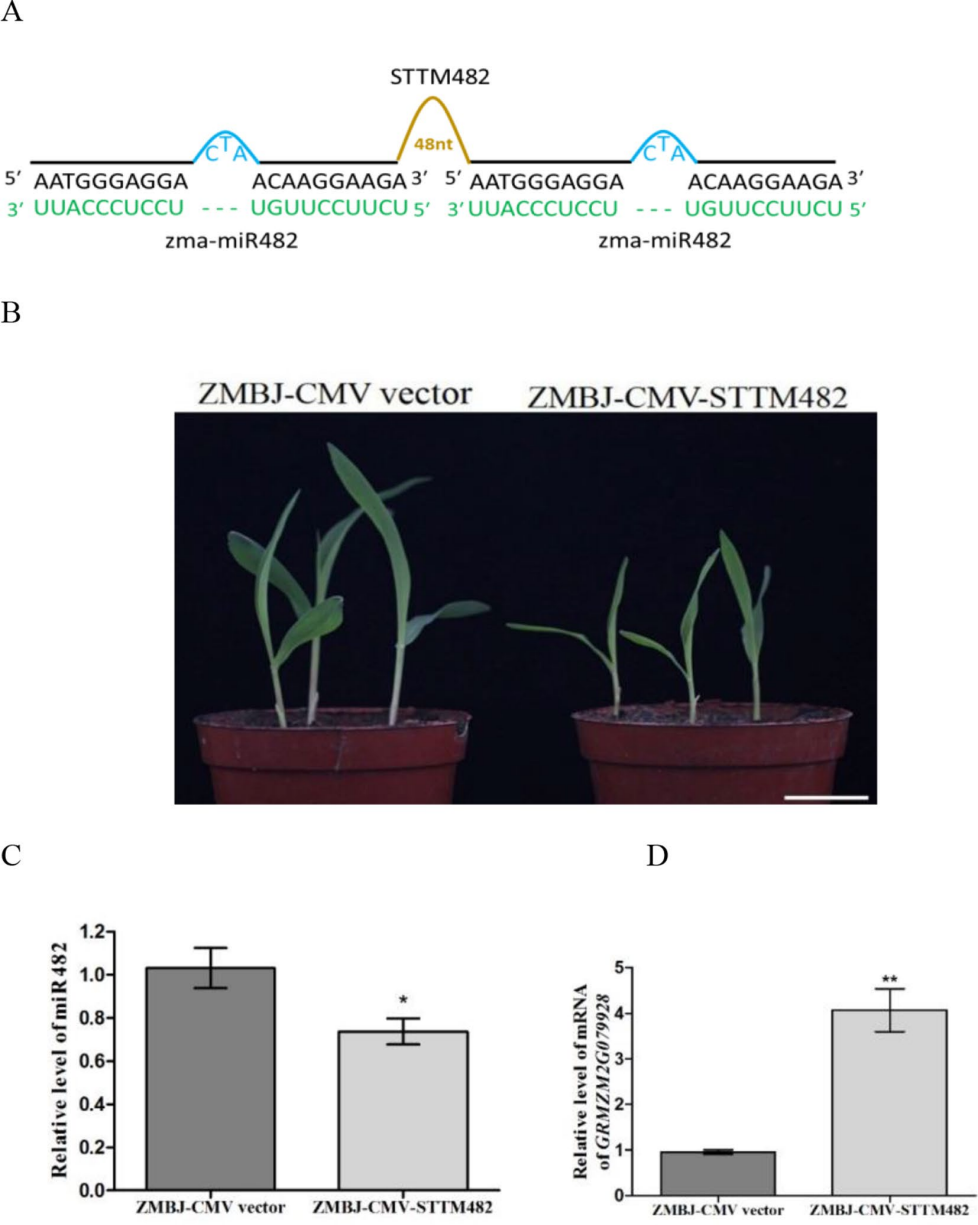
Silencing miR482 expression in maize using ZMBJ-CMV-STTM482. **(A)** a diagram of the STTM482 insert in the ZMBJ-CMV-based VbMS vector. 48 nt, 48-nt long stem-loop like linker; —, no nt at this position. **(B)** Maize seedlings infected with ZMBJ-CMV (control) or ZMBJ-CMV-STTM482 were photographed at 14 dpi. Bars = 2 cm. **(C)** Relative expression of miR482 in maize was determined by stem-loop RT-qPCR. **(D)** Relative expression of miR482 target gene GRMZM2G079928 in the ZMBJ-CMV or ZMBJ-CMV-STTM482-infected plants. The first true leaves of the assayed seedlings were harvested at 14 dpi and used for this study. The results were analyzed and presented as described in **Figure 2**. *, p < 0.05; **, p < 0.01.

miR482 is first identified in populus (*Populus trichocarpa*) as a novel and wounding-responsive microRNA (Lu et al., 2005), and can regulate the expression of disease resistance protein genes in populus, soybean and tomato (Lu et al., 2008; Li et al., 2010; Shivaprasad et al., 2012). In this study, we analyzed the role of miR482 in maize using vector ZMBJ-CMV-STTM482. Results showed that the control plants infected with ZMBJ-CMV did not show clear developmental defects. In contrast, the plants infected with ZMBJ-CMV-STTM482 showed retarded growth with shorter stem node and narrower leaves (**Figures 5A, B**). These phenotypes suggested that suppression of miR482 expression could inhibit maize growth. Results of RT-qPCR showed that the relative expression level of miR482 was significantly down-regulated in the ZMBJ-CMV-STTM482-inoculated plants (**Figure 5C**). In contrast, the relative expression level of *ZmTPS2* (GRMZM2G079928, a predicted target gene of miR482) was significantly up-regulated (**Figure 5D**).

## Discussion

In this study, we reported a newly constructed ZMBJ-CMV-2b_N81_ vector and its application in miRNA loss-of-function studies in both *N. benthamiana* and maize plants. To the best of our knowledge, this ZMBJ-CMV-based VbMS system is currently the only system that can be used to study miRNA functions in maize plants. Because this VbMS system causes mild disease symptoms in *N. benthamiana* and maize plants, we were able to use the ZMBJ-CMV-STTM165/166 or ZMBJ-CMV-STTM159 vector to determine the functions of nbe-miR165/166 or nbe-miR159 in *N. benthamiana*. To confirm the usefulness of this system in maize, we inoculated the ZMBJ-CMV-STTM167 or ZMBJ-CMV-STTM482 vector to maize seeds through VPI to investigate the functions of zma-miR167 or zma-miR482 in maize studies. In all experiments, the four tested vectors induced specific and strong silencing of the target miRNAs, leading to visible and specific miRNA lost-of-function phenotypes. In addition, after silencing the target miRNAs, we found that the expression levels of miRNA target genes were upregulated significantly. We have demonstrated that insertion of STTM cassettes behind the truncated 2b gene lead to more stable expressions of STTM inserts (**Figure 1A**), resulting to stronger down-regulations of target miRNAs. Based on these results, we consider that this ZMBJ-CMV-based VbMS system can be a useful tool for reliable and high-throughput investigations of miRNA functions in maize.

In this study, all the *N. benthamiana* plants inoculated with the ZMBJ-CMV-STTM165/166 exhibited ectopic leaf outgrowths on the abaxial leaf side (**Figure 2**). Also, all the *N. benthamiana* plants inoculated with the ZMBJ-CMV-STTM159 showed strong plant dwarfing and leaf malformation phenotypes by 1 week post virus inoculation (**Figure 3**). These phenotypes resembled that reported in previous reports (Liao et al., 2015; Zhao et al., 2016). It is noteworthy that the defective phenotypes caused by the downregulation of miRNA165/166 using the system described here were less strong than that reported previously using TRV, LS-CMV and PVX systems (Sha et al., 2014; Liao et al., 2015; Zhao et al., 2016). We speculate that this difference is likely caused by different virulence of different viruses or virus strains. Because the ZMBJ-CMV-based STTM system induces mild disease symptoms, it may not induce strong miRNA165/166 knockdown phenotypes.

It was reported that miR167 could regulate Arabidopsis and soybean lateral roots development (Gifford et al., 2008; Wang et al., 2015). Arabidopsis *MIM167* mutant plants were reported to show delayed flowering and distorted leaf phenotypes (Todesco et al., 2010). For soybean, the miR167c-overexpressing roots had much more lateral roots and longer length than that shown by the wild type plants. In contrast, transgenic soybean roots with reduced level of miR167 level showed a substantial decrease of lateral root numbers and length compared with the empty vector transformed soybean roots (Wang et al., 2015). Results obtained in this study also confirm that suppression of miR167 expression in maize by ZMBJ-CMV-STTM167 resulted in the decrease of lateral root number and length comparing with that shown by the CMV empty vector-infected maize roots (**Figure 4**).

Members in the miR482/2118 family form a distinct class of 22-nt miRNAs and are partially overlapped. miR482/2118 were predicted to target conserved sequences encoding the P-loop motif in the NBS-LRR receptors (Shivaprasad et al., 2012). Members in the miR482 subfamily were reported to target NLR mRNAs and to trigger productions of secondary siRNAs. A recent report showed that STTM482 transgene tomato lines displayed enhanced resistance to pathogenic oomycete and bacteria (Canto-Pastor et al., 2019). However, the effects of miR482 on plant development and growth are unknown. In this study, silencing miR482 expression by the ZMBJ-CMV VbMS system up-regulated the expression of *ZmTPS2* (GRMZM2G079928), leading to a delayed growth of maize seedling (**Figure 5**). Trehalose-6-Phosphate Synthase (TPS) is a glycosyltransferase that catalyzes the synthesis of alpha, alpha-1,1-trehalose-6-phosphate from glucose-6-phosphate using UDP-glucose as a donor. In tobacco (*N. tabacum*), expression of *Escherichia coli* TPS (OTS A) results in an increase of photosynthetic activity, stunted plant growth and formation of lancet-shaped leaves (Paul et al., 2001; Pellny et al., 2004). The delayed growth of the ZMBJ-CMV-STTM-miR482 infected maize seedlings may be caused by the upregulation of *ZmTPS2* expression.

In conclusion, the results presented here show that the newly developed ZMBJ-CMV-based VbMS system can be used to dissect the functions of uncharacterized maize miRNAs. The efficiency of miRNA downregulation by this system is high enough to induce specific phenotypes in maize. Because ZMBJ-CMV can infect over 25 maize inbred lines with important breeding values (Wang et al., 2016), this new ZMBJ-CMV-based VbMS system should have the potential for the investigatation of miRNA in important maize breeding lines.

We consider that further optimizations can still be made to this VbMS system, in order to achieve a stable and long lasting miRNA suppression in mature maize plants. For example, the current VPI inoculation can be difficult for new researchers and may cause severe mechanical damages to embryos, leading to death or poor seed germinations. We consider that agro-inoculation of this VbMS vector to maize root system should be tested in our future experiment. Second, direct inoculation of VbMS vector to young corn ears or tassels may allow identifications of miRNAs important for maize propagative tissue growth.

## Data Availability Statement

The datasets generated for this study are available on request to the corresponding author.

## Author Contributions

XL and TZ conceived the study. XL, SL, RW, and XC performed the experiments and analyzed the data. XL, ZF, BW, and TZ wrote the manuscript. All authors approved the final version of the manuscript.

## Funding

This work was supported by grants from the National Natural Science Foundation of China (Grant 31570141), the Ministry of Agriculture and Rural Affairs of China (2018YFD020062, 2016ZX08010-001), and the Ministry of Education of China (the 111 Project B13006).

## Conflict of Interest

The authors declare that the research was conducted in the absence of any commercial or financial relationships that could be construed as a potential conﬂict of interest.
